# Geriatric End-of-Life Screening Tool Prediction of 6-Month Mortality in Older Patients

**DOI:** 10.1001/jamanetworkopen.2024.14213

**Published:** 2024-05-31

**Authors:** Adrian D. Haimovich, Ryan C. Burke, Larry A. Nathanson, David Rubins, R. Andrew Taylor, Erin K. Kross, Kei Ouchi, Nathan I. Shapiro, Mara A. Schonberg

**Affiliations:** 1 Department of Emergency Medicine, Beth Israel Deaconess Medical Center, Boston, Massachusetts; 2 Department of Internal Medicine, Beth Israel Deaconess Medical Center, Boston, Massachusetts; 3 Department of Emergency Medicine, Yale School of Medicine, New Haven, Connecticut; 4 Division of Pulmonary, Critical Care, and Sleep Medicine, University of Washington, Seattle; 5 Cambia Palliative Care Center of Excellence at UW Medicine, Seattle, Washington; 6 Department of Emergency Medicine, Brigham and Women’s Hospital, Boston, Massachusetts

## Abstract

**Importance:**

Emergency department (ED) visits by older adults with life-limiting illnesses are a critical opportunity to establish patient care end-of-life preferences, but little is known about the optimal screening criteria for resource-constrained EDs.

**Objectives:**

To externally validate the Geriatric End-of-Life Screening Tool (GEST) in an independent population and compare it with commonly used serious illness diagnostic criteria.

**Design, Setting, and Participants:**

This prognostic study assessed a cohort of patients aged 65 years and older who were treated in a tertiary care ED in Boston, Massachusetts, from 2017 to 2021. Patients arriving in cardiac arrest or who died within 1 day of ED arrival were excluded. Data analysis was performed from August 1, 2023, to March 27, 2024.

**Exposure:**

GEST, a logistic regression algorithm that uses commonly available electronic health record (EHR) datapoints and was developed and validated across 9 EDs, was compared with serious illness diagnoses as documented in the EHR. Serious illnesses included stroke/transient ischemic attack, liver disease, cancer, lung disease, and age greater than 80 years, among others.

**Main Outcomes and Measures:**

The primary outcome was 6-month mortality following an ED encounter. Statistical analyses included area under the receiver operating characteristic curve, calibration analyses, Kaplan-Meier survival curves, and decision curves.

**Results:**

This external validation included 82 371 ED encounters by 40 505 unique individuals (mean [SD] age, 76.8 [8.4] years; 54.3% women, 13.8% 6-month mortality rate). GEST had an external validation area under the receiver operating characteristic curve of 0.79 (95% CI, 0.78-0.79) that was stable across years and demographic subgroups. Of included encounters, 53.4% had a serious illness, with a sensitivity of 77.4% (95% CI, 76.6%-78.2%) and specificity of 50.5% (95% CI, 50.1%-50.8%). Varying GEST cutoffs from 5% to 30% increased specificity (5%: 49.1% [95% CI, 48.7%-49.5%]; 30%: 92.2% [95% CI, 92.0%-92.4%]) at the cost of sensitivity (5%: 89.3% [95% CI, 88.8-89.9]; 30%: 36.2% [95% CI, 35.3-37.1]). In a decision curve analysis, GEST outperformed serious illness criteria across all tested thresholds. When comparing patients referred to intervention by GEST with serious illness criteria, GEST reclassified 45.1% of patients with serious illness as having low risk of mortality with an observed mortality rate 8.1% and 2.6% of patients without serious illness as having high mortality risk with an observed mortality rate of 34.3% for a total reclassification rate of 25.3%.

**Conclusions and Relevance:**

The findings of this study suggest that both serious illness criteria and GEST identified older ED patients at risk for 6-month mortality, but GEST offered more useful screening characteristics. Future trials of serious illness interventions for high mortality risk in older adults may consider transitioning from diagnosis code criteria to GEST, an automatable EHR-based algorithm.

## Key Points

**Question:**

Can the Geriatric End-of-Life Screening Tool (GEST) accurately identify older adults in the emergency department with high 6-month mortality risk?

**Findings:**

In this prognostic study of 82 371 ED encounters within a tertiary care emergency department, GEST performed robustly on external validation, identifying 11.6% of the population as having a 30% or greater mortality risk. Compared with serious illness diagnoses, GEST provided a greater net benefit as a screening tool using decision curve analysis.

**Meaning:**

The findings of this prognostic external validation study highlight the opportunity to use pragmatic, prognostic electronic health record algorithms to identify older adults in the emergency department for end-of-life care interventions.

## Teaser

This prognostic study examines the Geriatric End-of-life Screening Tool for use in the evaluation of mortality risk in older patients presenting to the emergency department.

## Introduction

Emergency departments (EDs) are a vital setting in which to deliver goal-concordant end-of-life care. First, approximately 75% of adults aged 65 years or older will present to an ED within 6 months of dying.^1^ Second, clinical decisions made in the ED have profound roles in patient health care trajectories.^2,3^ Often, ED clinicians must make clinical recommendations and decisions with limited knowledge of the longitudinal course of a patient’s condition. Advance care planning documentation is rarely available to inform these ED processes^4^ and, when available, may be outdated.^5,6^ For these reasons, incorporating patient preferences is critical for patient-centered ED care. Despite this, few ED physicians report incorporating patient values and goals into treatment decisions, instead focusing primarily on procedures (eg, intubation) and processes (eg, admission).^7^

Closing this care gap requires the development of scalable and useful interventions designed to support ED clinicians first eliciting patient goals for care and then integrating these goals into the medical decision-making process. While proactive patient identification is a key facilitator to the success of these interventions,^8^ there is a lack of pragmatic tools to identify the patients most likely to benefit. Numerous screening instruments to identify at-risk older adults have been proposed, including the Identification of Seniors at Risk,^9^ the Triage Risk Screening Tool,^10^ the Screening Tool Risk Score Assessment,^11^ and the Surprise Question,^12^ but all require data often unavailable in the electronic health record (EHR) (eg, specific survey responses from the patients or clinicians), limiting their applicability. As a result, only approximately 10% of ED clinicians report using an successful strategy to screen and refer patients to palliative care.^13,14^

The use of diagnosis codes to identify patients with life-limiting illnesses is increasingly common.^15,16^ While efforts to operationalize these definitions for the purposes of prospective patient identification have yielded variable patient inclusion criteria between studies, commonly used diagnoses include metastatic cancers, dementia, severe chronic obstructive pulmonary disease, diabetes with complications, congestive heart failure, and neurodegenerative diseases.^17,18^ While benefiting from simplicity of implementation as EHR-based diagnosis codes are ubiquitously available, this approach to serious illness identification is limited in both sensitivity and specificity. First, serious illnesses criteria will miss acutely ill patients if they do not have an underlying serious illness diagnosis code. Second, while these diagnoses are associated with a higher mortality, they do not enable further risk stratification between patients with potentially stable vs unstable disease.

Mortality risk screening using automatable EHR algorithms is an attractive alternative to the use of serious illness diagnosis codes. An internally validated prognostic logistic regression algorithm was developed specifically for the ED setting: the Geriatric End-of-Life Screening Tool (GEST). GEST was derived using data from 9 EDs in a large Connecticut-based health system and uses readily available EHR variables, including age, vital signs, blood counts, past medical diagnoses, and hospitalizations within the past year to estimate 6-month mortality of ED patients aged 65 years or older.^19^ The study reported that GEST has robust discrimination and calibration across race and ethnicities, as well as age deciles.^19^ GEST significantly outperformed both the Charlson and Elixhauser comorbidity indices in identifying high-risk older adults.

Recognizing that clinical prediction models often experience substantial degradation in model performance on external validation,^20,21,22^ in this study we externally validated GEST at a large, Boston-based tertiary care ED and assessed model performance over time and across demographic subgroups. We then used GEST to investigate the distribution of 6-month mortality risk of older adults in the ED with and without serious illnesses to identify potentially misclassified serious illness populations, namely high-risk older adults without serious illness who may benefit from serious illness interventions and, conversely, low-risk older adults with serious illnesses who may be less likely to benefit.

## Methods

### Study Setting and Participants

This prognostic retrospective observational cohort study included all adults aged 65 years or older who presented to the Beth Israel Deaconess Medical Center ED between 2017 and 2021 and had a residential address in Massachusetts. The study was deemed exempt by the Beth Israel Deaconess Medical Center Institutional Review Board as secondary research for which consent is not required. We excluded encounters presenting with cardiac arrest and encounters where death occurred within 1 day of ED arrival.^19^ We followed the Transparent Reporting of a Multivariable Prediction Model for Individual Prognosis or Diagnosis (TRIPOD) reporting guideline.^23^

### Variables and Measures

All patient-level data, including race and ethnicity for demographic reporting, were extracted from the institutional EHR. Use of the institutional EHR is limited to the Beth Israel Deaconess Medical Center campus and a number of associated outpatient clinical practices.

### Outcome

Our primary outcome was mortality at 6 months to align with the design of the GEST derivation study. We combined EHR-based mortality records with Massachusetts state vital records through December 2022 (eMethods 1 in Supplement 1). Because a single individual’s mortality risk may vary between ED visits, our unit of analysis is at the level of the encounter, rather than patient, and a single patient may have multiple encounters in our study.

### GEST External Validation

A logistic regression based predictive algorithm, GEST uses the following covariates: age, hemoglobin level, hematocrit level, blood urea nitrogen level, reticulocyte distribution width, lymphocyte count, mean corpuscular volume, mean heart rate, minimum systolic blood pressure, use of supplemental oxygen in the ED, number of admissions in the past year, history of secondary cancer, delirium, dementia or other cognitive disorders, lung cancer, pancreatic cancer, ED diagnosis of syncope or acute cerebrovascular disease, and presence of outpatient cardiovascular medications (eTable 1 and eTable 2 in Supplement 1).^19^ For vital signs, we used only the first 4 hours of the ED visit. We used the missing value imputation, standardization, and logistic regression values as described in the original study (eMethods 2 and eTable 3 in Supplement 1). Outpatient medications were not available in this external validation cohort and these missing values were imputed using the median value from the GEST derivation study (eTable 3 in Supplement 1). All years were used for these analyses (2017-2021).

We assessed the discrimination of GEST on external validation using area under the receiver-operating characteristic curves (AUROCs) with 95% CIs from the DeLong method.^24^ For calibration, we divided encounters in the validation cohort into risk groups based on their GEST-assigned probability and evaluated their observed mortality rates. In alignment with current recommendations designed to mitigate algorithmic bias, we repeated the AUROC analysis substratified by gender and race and ethnicity.^25,26^ Due to limited sample size, our racial subgroup analysis only included Asian, Black, and White patients. We evaluated GEST performance over time by comparing results from each study year.

### GEST Model Update

Prior research has shown that clinical risk models often perform poorly on external validation^27^ and can benefit from updating methods before implementation in a new setting. We tested the impact of updating GEST via recalibration (eMethods 3 in Supplement 1) and refitting (eMethods 4 in Supplement 1).^28^ Data from 2017-2018 were used for model modification and the years 2019-2021 were used for validation of the modified models. We compared performance of the modified models with the original GEST model using AUROCs and calibration plots from the validation dataset.

### Serious Illness Criteria

For serious illnesses, we used *International Statistical Classification of Diseases and Related Health Problems, 10th Revision*, codes for stroke/transient ischemic attack, liver disease, hip fracture, cancer, heart disease, lung disease, neurodegenerative disease, diabetes with associated peripheral vascular disease, coronary artery disease, chronic kidney disease, HIV/AIDS, kidney failure, or dementia (eMethods 5 in Supplement 1 and the eTable in Supplement 2).^16^ Only a single serious illness was required to be included in the serious illness group.^17^ We used a 1-year lookback period from each index ED visit to identify these codes. As the serious illnesses classification is binary, no AUROC can be calculated. In alignment with other studies, we included age 80 years or older as a serious illness.^17^

### Statistical Analysis

Data analysis was performed from August 1, 2023, to March 27, 2024. We performed a 6-month survival analysis for both GEST risk groups for serious illness screening. For this analysis, we used only the first encounter for a given patient during the study period. To illustrate a range of GEST risks, we identified groups of patients with greater than 5%, 10%, 20%, and 30% mortality risk. We right-censored all visits without known mortality on December 31, 2022, to match our most recent state-level mortality data. Kaplan-Meier estimators were developed with the Python, version 3.10.4 *statsmodels* library (Python Software Foundation).

We calculated sensitivity, specificity, positive and negative predictive values, positive and negative likelihood ratios, and the total proportion of study encounters that would screen positive for GEST using 5%, 10%, 20%, and 30% risk cutoffs, comparing these measures with encounters with and without serious illness criteria. As a sensitivity analysis to account for patient clustering, we repeated these calculations when including only the first ED visit per patient in the study period. As a second sensitivity analysis, we repeated this analysis using serious illness diagnoses without age criteria.

To characterize misclassified patients based on serious illness criteria, we identified encounters without serious illness criteria with patients who had a greater than 30% mortality risk and patients with serious illness criteria who had a less than 10% mortality risk. We performed decision curve analysis comparing the original GEST algorithm^19^ with serious illness screening across a range of mortality risk thresholds. We used a significance threshold of .05, where relevant and testing was 2-sided and unpaired.

## Results

After excluding 49 visits with a chief presentation of cardiac arrest and 280 visits with death within 1 day of ED arrival, this study included 82 371 ED encounters by 40 505 patients with a mean (SD) age of 76.8 (8.4) years; 54.3% were female, 45.7% were male, 4.3% were Asian, 19.9% were Black, 6.7% were Hispanic, and 69.7% were White (Table 1). The 6-month mortality rate across this cohort was 13.8% (minimum, 12.9% in 2017; maximum, 15.1% in 2020). A total of 53.4% of patients met serious illness criteria. The most prevalent serious illnesses criteria were age 80 years or older (35.6%), heart disease (14.3%), and lung disease (12.6%), while the least prevalent were hip fracture (0.6%) and neurodegenerative disease (0.1%). Patients aged 80 years or older had an observed mortality rate of 19.5%. We report missing value information across the overall cohort in eTable 4 in Supplement 1.

**Table 1.** Patient Characteristics

Characteristic	No. (%)
No.	82 371
Age, mean (SD), y	76.8 (8.4)
Sex	
Female	44 747 (54.3)
Male	37 624 (45.7)
Ethnicity	
Hispanic or Latino	5549 (6.7)
Not Hispanic or Latino	74 949 (91.0)
Unknown	1873 (2.3)
Race	
Asian	3517 (4.3)
Black	16 375 (19.9)
Native American/Alaska Native	180 (0.2)
Native Hawaiian or other Pacific Islander	67 (0.1)
White	57 378 (69.7)
Unknown	1516 (1.8)
Other^a^	3338 (4.1)
ED disposition	
Admitted	45 932 (55.8)
Home	26 169 (31.8)
Observation then admitted	2649 (3.2)
Observation then home	7621 (9.3)
6-mo mortality	11 388 (13.8)
Serious illnesses	43 977 (53.4)
Dementia	3735 (4.5)
Kidney failure	2169 (2.6)
Diabetes	6018 (7.3)
Neurodegenerative disease	53 (0.1)
Lung disease	10 351 (12.6)
Heart disease	11 760 (14.3)
Cancer	4404 (5.3)
Hip fracture	512 (0.6)
Liver disease	2051 (2.5)
Stroke/TIA	1445 (1.8)
Age ≥80 y	29 346 (35.6)

Abbreviations: ED, emergency department; TIA, transient ischemic attack.

^a^ The Other racial group represents patients whose electronic health record racial category is Other. Observation was based in the ED, rather than inpatient records.

**Figure 1.**
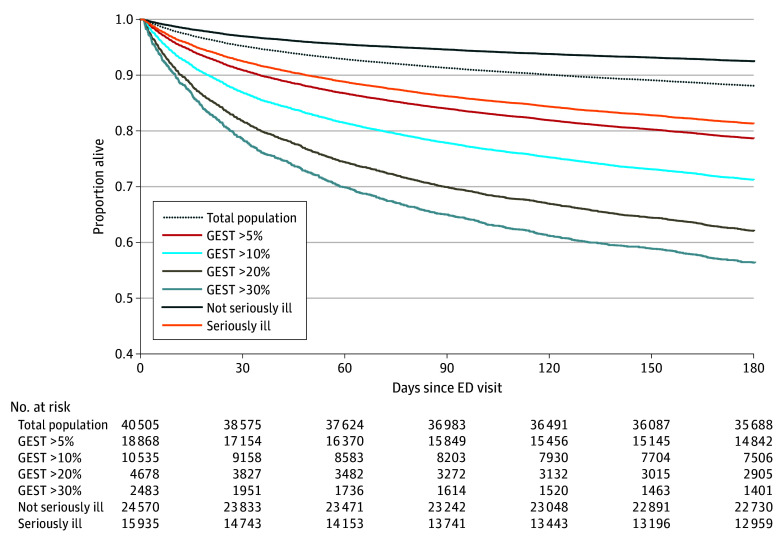
Survival Curves for 6 Months Following the First Emergency Department (ED) Encounter of Each Patient in the Study Period Total population includes all patients, regardless of serious illness diagnoses or Geriatric End-of-Life Screening Tool (GEST) risk. GEST curves include patients whose first encounter during the study period had a mortality risk greater than the indicated number.

In this external validation, GEST had an AUROC of 0.79 (95% CI, 0.78-0.79) (Figure 1A). Performance of GEST was stable across demographic groups (female: 0.80 [95% CI, 0.79-0.81], Hispanic: 0.78 [95% CI, 0.75-0.80], and Black: 0.82 [95% CI, 0.81-0.83]) and across age deciles (60s: 0.80 [95% CI, 0.80-0.82], 70s: 0.79 [95% CI, 0.79-0.80], 80s: 0.74 [95% CI, 0.73-0.75], and >90: 0.68 [95% CI, 0.67-0.70]). Discrimination of GEST by calendar year ranged from 0.78 (95% CI, 0.77-0.79) in 2019 to 0.80 (95% CI, 0.79-0.81) in 2021. We did not observe significant differences in GEST AUROC when considering only the first ED visit by each patient during the study period (eTable 5 in Supplement 1). We did not obtain significant improvements in discrimination (eFigure 1 in Supplement 1) or calibration (eFigure 2 in Supplement 1) by either updating the logistic regression intercept or refitting the logistic regression. Demographic characteristic details for this analysis are in eTable 6 in Supplement 1, and updated coefficients for the refit logistic regression model are reported in eTable 7 in Supplement 1. Because we did not observe significant improvements, we used the original GEST model for the remainder of our analyses and included patient encounters between 2017 and 2021.

Kaplan-Meier analyses of the first visit for each patient within the study period and stratified by GEST risk groups and serious illnesses are shown in Figure 1. The observed mortality rate of encounters with serious illness was 18.7% compared with 7.5% without. Encounters with a GEST risk greater than 5% had an observed mortality rate of 22.0%, while encounters with risk greater than 10% had mortality of 28.8%; greater than 20%, 38.0%; and greater than 30%, 43.7%.

**Figure 2.**
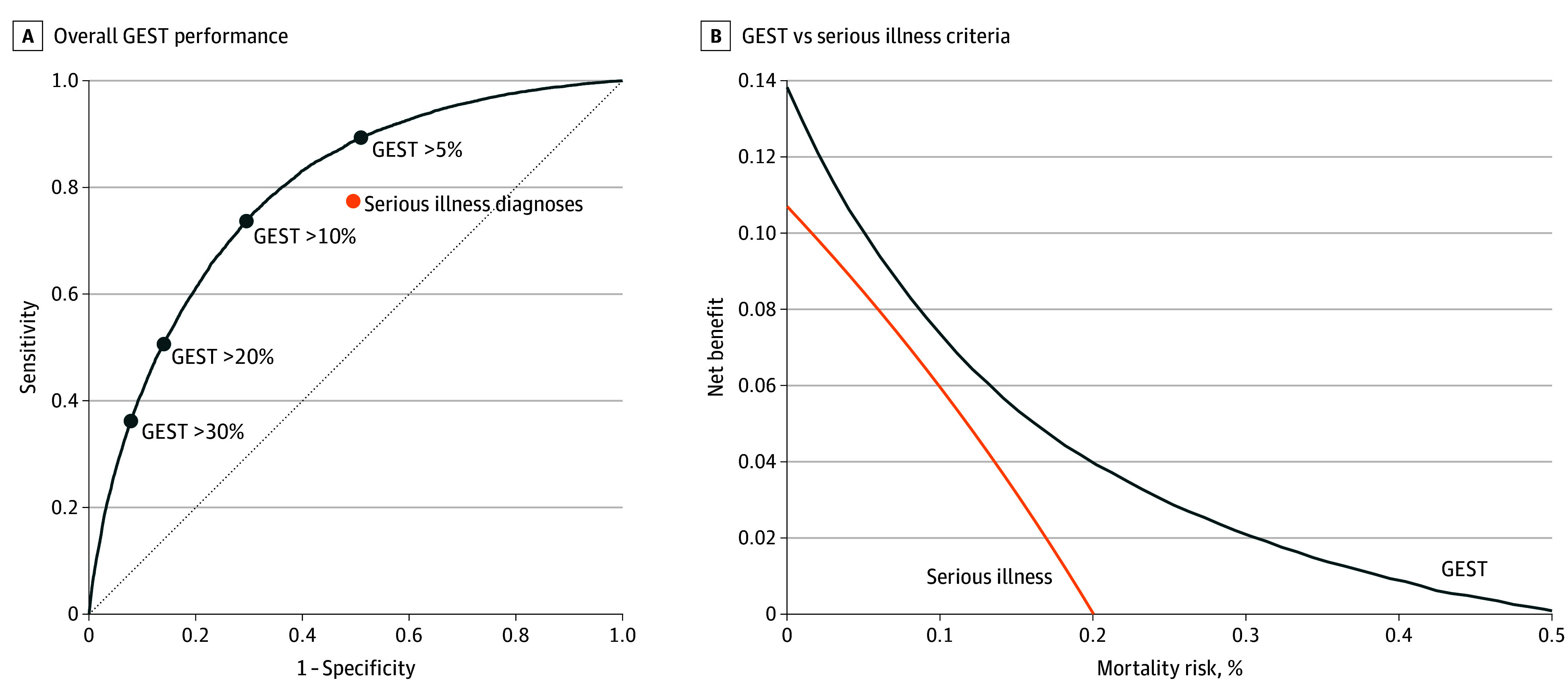
Performance of Geriatric End-of-Life Screening Tool (GEST) Compared With Serious Illness Criteria A, GEST performance across all sensitivities and specificities is shown as a receiver-operating characteristic curve. Because serious illness criteria are binary, only a single sensitivity and specificity value is shown. B, Decision curve analysis comparing GEST with serious illness criteria. In decision curve analysis of a screening test, the x-axis is the threshold at which screening would be indicated. The y-axis is net benefit defined as true-positives minus false-positives times the exchange rate, where the exchange rate is a function of the x-axis threshold probability. The greatest net benefit screening tool can be found by identifying the highest line on the y-axis for a given mortality risk threshold on the x-axis.

Across all ED encounters, 53.4% involved patients with serious illnesses, with a sensitivity of 77.4% (95% CI, 76.6%-78.2%) and specificity 50.5% (50.1%-50.8%) for 6-month mortality (Table 2, Figure 2A). The trade-offs between sensitivity and specificity for GEST at various thresholds are shown in a receiver-operating characteristic curve (Figure 2). A GEST cutoff of 5% included 56.2% of patients with a sensitivity of 89.3% (95% CI, 88.8%-89.9%) and specificity of 49.1% (95% CI, 48.7%-49.5%), while cutoffs of 20% and 30% included 19.1% and 11.8% of all encounters, with sensitivities of 50.6% (95% CI, 49.7%-51.5%) and 36.2% (95% CI, 35.3%-37.1%) and specificities of 86.0% (95% CI, 85.7%-86.2%) and 92.2% (95% CI, 92.0%-92.4%). Using only the first ED visit for a given patient during the study period significantly lowered sensitivity and increased specificity for both serious illness criteria and the GEST thresholds identified (eTable 8 in Supplement 1). Similarly, removing age criteria from serious illness definitions decreased the percent of identified patients to 28.4%, concurrently increased specificity to 74.7% (95% CI, 74.4%-75.0%), and decreased sensitivity to 47.8% (95% CI, 46.9%-48.7%) (eFigure 3 in Supplement 1).

**Table 2.** Test Characteristics of GEST Compared With Serious Illness Diagnoses for All Encounters in the Study Period

Characteristic	Patients, %^a^	% (95% CI)		PPV (95% CI)	NPV (95% CI)	LR^+^ (95% CI)	LR^−^ (95% CI)
		Sensitivity	Specificity				
Serious illness	53.4	77.4 (76.6-78.2)	50.5 (50.1-50.8)	20.0 (20.0-20.0)	93.3 (93.0-94.0)	1.6 (1.5-1.6)	0.45 (0.43-0.47)
GEST >5%	56.2	89.3 (88.8-89.9)	49.1 (48.7-49.5)	22.0 (22.0-22.0)	96.6 (96.0-97.0)	1.8 (1.7-1.9)	0.22 (0.21-0.23)
GEST >10%	35.6	73.7 (72.9-74.5)	70.5 (70.2-70.8)	28.6 (28.0-29.0)	94.3 (94.0-95.0)	2.5 (2.4-2.6)	0.37 (0.36-0.39)
GEST >20%	19.1	50.6 (49.7-51.5)	86.0 (85.7-86.2)	36.6 (36.0-37.0)	91.6 (91.0-92.0)	3.6 (3.5-3.7)	0.57 (0.55-0.6)
GEST >30%	11.8	36.2 (35.3-37.1)	92.2 (92.0-92.4)	42.5 (42.0-44.0)	90.0 (90.0-90.0)	4.6 (4.4-4.8)	0.69 (0.66-0.72)

Abbreviations: GEST, Geriatric End-of-Life Screening Tool; LR^+^, positive likelihood ratio; LR^−^, negative likelihood ratio; NPV, negative predictive value; PPV, positive predictive value.

^a^ Percent of patients indicates the percentage of all encounters meeting the criteria.

We performed a decision curve analysis comparing GEST with serious illness criteria across the entire range of mortality risk thresholds (Figure 2B). In this analysis, the x-axis represents the mortality risk threshold at which an intervention (eg, patient discussion, palliative consult) would be indicated. The y-axis is the true-positives identified by the screening method minus the false-positives weighted by an exchange rate that is a function of the x-axis threshold.^19^ The preferred screening tool for a given mortality risk threshold is the one with the greatest net benefit (ie, higher y-axis line); GEST outperformed serious illness criteria across the range of thresholds.

**Figure 3.**
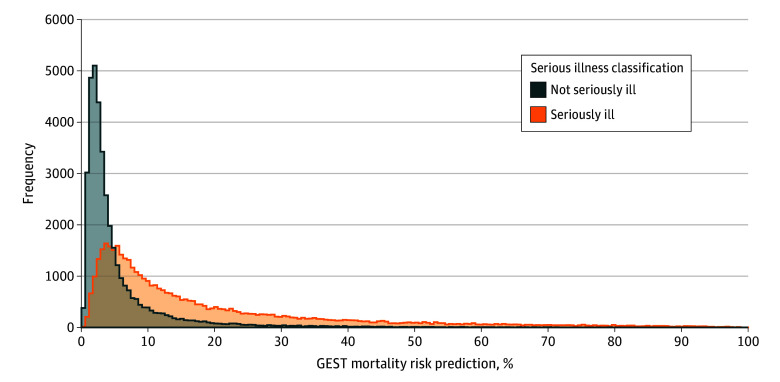
Distribution of Geriatric End-of-Life Screening Tool (GEST) Mortality Risk Across Patient Encounters With and Without Serious Illnesses Patient encounters were stratified by the presence or absence of serious illness diagnoses, including age criteria.

The median GEST mortality risk of patient encounters without serious illness was 3.1% (IQR, 1.9%-5.9%), while encounters of patients with serious illness had a median GEST risk of 11.5% (IQR, 5.7%-24.8%) (Figure 3). Of patients with serious illness, 45.1% were identified as low risk using GEST, with an observed mortality rate of 8.1% at 6 months, while 19.8% were high risk with an observed mortality rate of 43.5%. Of patients without serious illness, 86.5% were low risk by GEST mortality risk with an observed mortality rate of 4.2%, while 2.6% were high risk with an observed mortality rate of 34.3%. Overall, 25.3% of the encounters in this cohort had either serious illness with a low risk from GEST or did not have serious illness with a high risk from GEST.

## Discussion

In this retrospective prognostic cohort study, we externally validated the GEST screening instrument for identifying older ED patients with high short-term mortality risk, finding robust performance across gender, race and ethnicity, and chronological time. To our knowledge, this is the first example of an automatable end-of-life predictive algorithm being validated outside of the original derivation health system in the US.^29^ We note several differences between this external validation study and the GEST derivation study.^19^ First, the mortality rate in this study (13.8%) was higher than the derivation study mortality rate of 12.2%. Second, this cohort was slightly younger and had a higher percentage of patients of races and ethnicities other than White. Third, one of the model parameters, outpatient cardiovascular medications, was not available. Fourth, data for this study were derived from a different EHR system with a smaller user base, which may have affected diagnosis code capture. We hypothesize that the confluence of these features led to the modest decrease in discrimination in this external validation (AUROC, 0.79) compared with the derivation study (AUROC, 0.82).

We found that serious illness criteria captured a higher mortality risk population, but that 53.4% of all encounters in our cohort met these criteria, leading to lower specificity (50.5%). While low-resource interventions, such as EHR alerts,^17^ may be feasible for large populations with lower mortality risk, more specificity may be required to maximize the efficacy of high-resource interventions, such as palliative care consults. Varying the GEST threshold enables implementing EDs to balance sensitivity, specificity, and total volume of patients screening positive for subsequent intervention. In decision curve analysis, GEST outperformed serious illness criteria across the entire range of mortality thresholds, implying that, regardless of the choice of mortality risk cutoff to initiate serious illness interventions, GEST offered the greatest net benefit.

GEST offers several advantages compared with other serious illness algorithms. First, it is algorithmically tractable with only 18 covariates as opposed to hundreds or thousands in other approaches, reducing implementation and maintenance costs.^30^ Second, GEST uses vital signs and laboratory data typically available in the first few hours of an ED visit, while other mortality-based screening tools often require more than 12 hours of data prior to intervention.^31^ Third, GEST does not target a certain serious illness category, such as cancer, enabling screening across an undifferentiated patient population.^32^

Once locally validated, we envision 3 core clinical care pathways that can be implemented using GEST.^13^ First, GEST can be used to identify patients planned for admission for inpatient palliative care consultation^33^ or team-based serious illness discussions.^34^ Second, for patients planned for discharge,^35^ GEST can trigger communication interventions between the ED and primary care or direct engagement of outpatient palliative care. Third, for a subset of patients, serious illness interventions in the ED via an ED physician,^36,37^ nurse,^38^ social worker,^39^ or palliative care team member^40^ may change the trajectory of their care in the ED, especially in light of the accelerating ED boarding crisis.^41^ For example, hospice initiation from the ED can improve patient-centered outcomes while decreasing hospital resource use and length of stay.^42^ While the findings of a recent trial of an automated serious illness conversation suggest that serious illness interventions can narrow disparities among racially or ethnically minoritized patients,^17^ ongoing vigilance for bias in access, process completion, and impact will be needed to ensure health equity.^43^

### Limitations

This study has limitations. While we propose that GEST is a useful screening tool to identify older adults in the ED for serious illness communication, we recognize that mortality risk is not an exclusive indication for these discussions.^31,44,45^ Both the development and validation of GEST took place in the Northeast US, which may limit its generalizability to other regions. Limitations to our serious illness analyses include selection of diagnostic codes for inclusion, as well as the possibility of incomplete EHRs at our institution, both pragmatic restrictions that may affect serious illness screening criteria in other institutions as well. The EHR used in this study includes a single hospital, so the data contained within may have different missingness than EHRs from larger integrated EHRs. Moreover, as our goal was primarily validation rather than model enhancement, we did not consider the addition of new parameters to the GEST model.

## Conclusions

The findings of this prognostic model validation study suggest that GEST offers a robust, pragmatic, and fully automatable approach to identify older patients in the ED with high short-term mortality risk. Tethering this screening approach to communication interventions may facilitate goal-concordant care of older adults in the ED.

## Notes

Supplement 1. **eMethods.** Detailed Methods **eFigure 1.** Receiver-Operating Characteristic Curve for Recalibrated Models **eFigure 2.** Calibration Plot for Recalibrated Models **eFigure 3.** Comparison of GEST Receiver-Operating Characteristic Curve to Serious Illness Screening Without Age Criteria Point Estimate **eTable 1.** *ICD-9* Codes Used for GEST **eTable 2.** *ICD-10* Codes Used for GEST **eTable 3.** GEST Model Values **eTable 4.** Missing GEST Variables Across the Study Cohort **eTable 5.** GEST AUROCs for All Encounters vs First Index Encounter per Patient During Study Period **eTable 6.** Population Demographics for Model Update Procedures **eTable 7.** Comparison Between Original Logistic Regression Coefficients and Updated Coefficient Model **eTable 8.** Test Characteristics of GEST as Compared to Serious Illness Diagnoses for Only the First Encounter per Patient in the Study Period **eReference**s

Supplement 2. **eTable.** Serious Illness *ICD-10* Codes

Supplement 3.
Data Sharing Statement
