# Supplementary material for: Geriatric End-of-Life Screening Tool Prediction of 6-Month Mortality in Older Patients
Source: JAMA Netw Open. 2024 May 31;7(5):e2414213. doi: 10.1001/jamanetworkopen.2024.14213 (PMC11143461; doi:10.1001/jamanetworkopen.2024.14213)
Supplement: Supplement 1. — eMethods. Detailed Methods eFigure 1. Receiver-Operating Characteristic Curve for Recalibrated Models eFigure 2. Calibration Plot for Recalibrated Models eFigure 3. Comparison of GEST Receiver-Operating Characteristic Curve to Serious Illness Screening Without Age Criteria Point Estimate eTable 1. ICD-9 Codes Used for GEST eTable 2. ICD-10 Codes Used for GEST eTable 3. GEST Model Values eTable 4. Missing GEST Variables Across the Study Cohort eTable 5. GEST AUROCs for All Encounters vs First Index Encounter per Patient During Study Period eTable 6. Population Demographics for Model Update Procedures eTable 7. Comparison Between Original Logistic Regression Coefficients and Updated Coefficient Model eTable 8. Test Characteristics of GEST as Compared to Serious Illness Diagnoses for Only the First Encounter per Patient in the Study Period eReferences [file jamanetwopen-e2414213-s001.pdf]

Supplementary Online Content

Haimovich AD, Burke RC, Nathanson LA, et al. End-of-life screening tool to predict 6-month mortality of older patients in the emergency department. *JAMA Netw Open*. 2024;7(5):e2414213. doi:10.1001/jamanetworkopen.2024.14213

eMethods. Detailed Methods

eFigure 1. Receiver-Operating Characteristic Curve for Recalibrated Models

eFigure 2. Calibration Plot for Recalibrated Models

eFigure 3. Comparison of GEST Receiver-Operating Characteristic Curve to Serious Illness Screening Without Age Criteria Point Estimate

eTable 1. *ICD-9* Codes Used for GEST

eTable 2. *ICD-10* Codes Used for GEST

eTable 3. GEST Model Values

eTable 4. Missing GEST Variables Across the Study Cohort

eTable 5. GEST AUROCs for All Encounters vs First Index Encounter per Patient During Study Period

eTable 6. Population Demographics for Model Update Procedures

eTable 7. Comparison Between Original Logistic Regression Coefficients and Updated Coefficient Model

eTable 8. Test Characteristics of GEST as Compared to Serious Illness Diagnoses for Only the First Encounter per Patient in the Study Period

This supplementary material has been provided by the authors to give readers additional information about their work.

© 2024 Haimovich AD et al. *JAMA Network Open.*

eMethods. Detailed Methods

Methods S1: Mortality linkage between electronic health records and state records

To combine Massachusetts mortality data with electronic health record data, we used a fuzzy probabilistic framework (Python Record Linkage Toolkit). We first required that the last names and dates of birth matched identically. We subsequently matched by first name with a Jaro-Winkler distance of 0.95 and gender (exact). Where a middle initial was available, we preferentially took matches that included a matching middle initial.

Methods S2: Implementation of GEST

To validate GEST, we extracted data from our institutional EHR using the first four hours of vital signs as in the GEST derivation study and past medical history from any time prior to the ED visit. For ED and past historical diagnoses, we used both ICD-9 and ICD-10 codes (Table S1-2). Values were then scaled with mean subtracted and division by the sample variance using values from Table S3. Missing values were then imputed using the values from the GEST derivation study (Table S3). Finally, we used the logistic function to get the model predictions using coefficients and intercept from Table S3.

Methods S3: GEST recalibration

First, we performed a model recalibration using the method of Janssen et al.[1] In this process, all model coefficients are identical to those in the derivation study, but the intercept is updated using the mortality rate in the recalibration/refitting cohort. To update the intercept, we add a correction factor using based on the following formula:

$$\ln\left(\frac{\left(\frac{mortality\ rate}{1 - mortality\ rate}\right)}{\left(\frac{mean\ predicted\ risk}{1 - mean\ predicted\ risk}\right)}\right)$$

Data from 2017-2018 were used to recalibrate the model and data from 2019-2021 were used to validate the recalibrated model.

Methods S4: GEST refitting

As a second approach, we refitted the logistic regression coefficients and intercept. We used the same covariates as in the original GEST model except for outpatient cardiovascular medications which we did not have available in this external validation dataset and so were excluded. To refit the logistic regression, we followed the methods of our derivation study.[2] First, we used median value imputation to impute missing values. We then standardized the data before fitting the logistic regression. Data from 2017-2018 were used to refit the logistic regression model and data from 2019-2021 were used to validate (Table S5).

Methods S5: Serious illness detection

To identify patients with serious illnesses, we used ICD-10 codes from Kelley et al (Supplementary eTable).[3,4] We included diabetes only if codes for coronary artery disease, kidney disease, or peripheral

© 2024 Haimovich AD et al. *JAMA Network Open.*

vascular disease were present. We also included all patients aged 80 or older.[5] For all diagnosis codes, we used only inpatient diagnoses from the year prior to the index ED visit.

© 2024 Haimovich AD et al. *JAMA Network Open.*

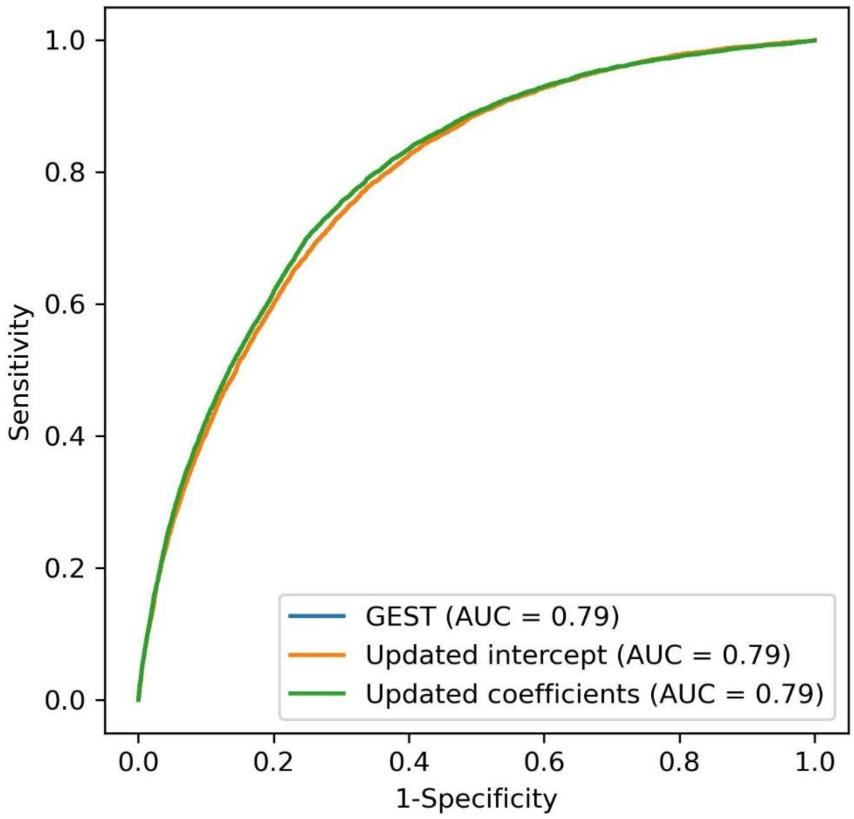

eFigure 1: Receiver-operating characteristic curve for recalibrated models. The GEST intercept (Updated intercept) or full model coefficients (Updated coefficients) were generated using data from 2017-2018 and then tested against data from 2019-2021. For this comparison, the original GEST coefficients (GEST) were also tested against the same data from 2019-2021.

© 2024 Haimovich AD et al. *JAMA Network Open.*

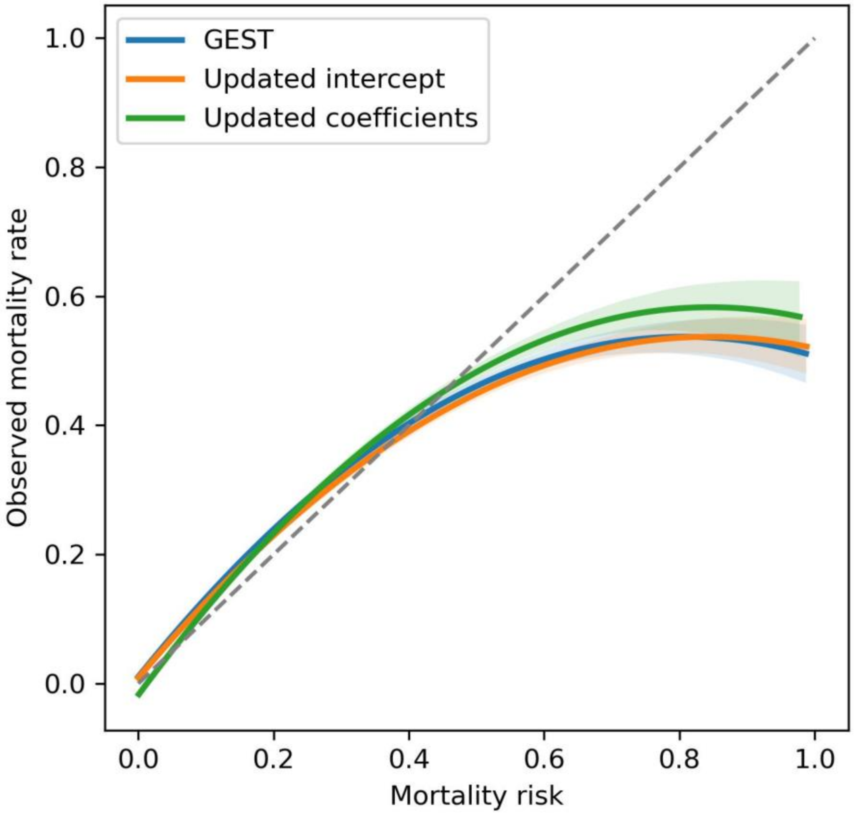

eFigure 2: Calibration plot for recalibrated models. The GEST intercept (Updated intercept) or full model coefficients (Updated coefficients) were recalibrated using data from 2017-2018 and then tested against data from 2019-2021. The original GEST coefficients (GEST) were also tested against data from 2019-2021.

© 2024 Haimovich AD et al. *JAMA Network Open.*

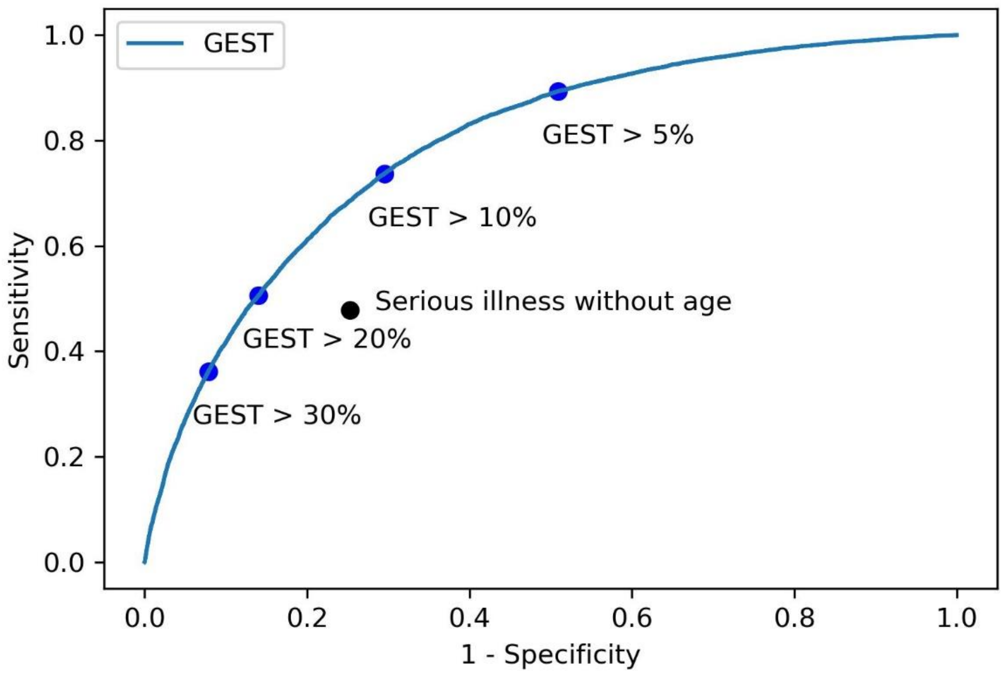

eFigure 3: Comparison of GEST receiver-operating characteristic curve to serious illness screening without age criteria point estimate. GEST performance is across all sensitivities and specificities is shown as a receiver-operating characteristic curve. GEST performance at selected cut-offs (5%, 10%, 20%, 30%) is shown with the identified points. As serious illness criteria are binary, only a single sensitivity and specificity is shown.

© 2024 Haimovich AD et al. *JAMA Network Open.*

eTable 1: ***ICD-9*** **codes used for GEST**

History of secondary malignancy	History of delirium, dementia, or other cognitive disorders	History of lung cancer	ED diagnosis of acute cerebrovascular disease	History of pancreatic cancer	ED diagnosis of syncope
1960	2900	1622	34660	1570	7802
1961	29010	1623	34661	1571	
1962	29011	1624	34662	1572	
1963	29012	1625	34663	1573	
1965	29013	1628	430	1574	
1966	29020	1629	431	1578	
1968	29021	20921	4320	1579	
1969	2903	2312	4321		
1970	29040	V1011	4329		
1971	29041		43301		
1972	29042		43311		
1973	29043		43321		
1974	2908		43331		
1975	2909		43381		
1976	2930		43391		
1977	2931		4340		
1978	2940		43400		
1980	2941		43401		
1981	29410		4341		
1982	29411		43410		
1983	29420		43411		
1984	29421		4349		
1985	2948		43490		
1986	2949		43491		
1987	3100		436		
19881	3102				
19882	3108				
19889	31081				
20971	31089				
20972	3109				
20973	3310				
20974	3311				
51181	33111				
78951	33119				
	3312				
	33182				
	797				

© 2024 Haimovich AD et al. *JAMA Network Open.*

eTable 2: ***ICD-10*** **codes used for GEST**

History of secondary malignancy	History of delirium, dementia, or other cognitive disorders	History of lung cancer	ED diagnosis of acute cerebrovascular disease	History of pancreatic cancer	ED diagnosis of syncope
C770	F0150	C33	G43.609	C250	R55
C771	F0151	C3400	G43.619	C251	
C772	F01511	C3401	G43.601	C252	
C773	F01518	C3402	G43.661	C253	
C774	F0152	C3410	I60.9	C254	
C775	F0153	C3411	I61.9	C257	
C778	F0154	C3412	I62.1	C258	
C779	F01A0	C342	I62.00	C259	
C7800	F01A11	C3430	I62.9		
C7801	F01A18	C3431	I63.22		
C7802	F01A2	C3432	I63.139		
C781	F01A3	C3480	I63.019		
C782	F01A4	C3481	I63.59		
C7830	F01B0	C3482	I63.20		
C7839	F01B11	C3490	I63.239		
C784	F01B18	C3491	I63.119		
C785	F01B2	C3492	I63.219		
C786	F01B3	C384	I66.09		
C787	F01B4	C390	I63.30		
C7880	F01C0	C399	I66.19		
C7889	F01C11	D021	I66.29		
C7900	F01C18	D0220	I63.50		
C7901	F01C2	D0221	I67.89		
C7902	F01C3	D0222	I63.40		
C7910	F01C4	D023	I66.9		
C7911	F0280	D024			
C7919	F0281				
C792	F02811				
C7931	F02818				
C7932	F0282				
C7940	F0283				
C7949	F0284				
C7951	F02A0				
C7952	F02A11				
C7960	F02A18				
C7961	F02A2				
C7962	F02A3				
C7963	F02A4				

C7970	F02B0
C7971	F02B11
C7972	F02B18
C7981	F02B2
C7982	F02B3
C7989	F02B4
C799	F02C0
	F02C11
	F02C18
	F02C2
	F02C3
	F02C4
	F0390
	F0391
	F03911
	F03918
	F0392
	F0393
	F0394
	F03A0
	F03A11
	F03A18
	F03A2
	F03A3
	F03A4
	F03B0
	F03B11
	F03B18
	F03B2
	F03B3
	F03B4
	F03C0
	F03C11
	F03C18
	F03C2
	F03C3
	F03C4
	F04
	F05
	F0670
	F0671
	F0781
	F0789

© 2024 Haimovich AD et al. *JAMA Network Open.*

F482
G300
G301
G308
G309
G3101
G3109
G311
G3183

© 2024 Haimovich AD et al. *JAMA Network Open.*

eTable 3: GEST model values

variable	mean	Standard deviation	imputed value	coefficient
Age	78.06858	79.759	-0.11965	0.508937
History of secondary malignancy	0.017583	0.017274	-0.13378	0.2183
History of lung cancer	0.024936	0.024315	-0.15992	0.138943
History of delirium, dementia, or other cognitive disorders	0.096954	0.087554	-0.32766	0.142679
History of pancreatic cancer	0.004482	0.004462	-0.0671	0.119607
Number of admissions in last year	0.938833	3.209384	-0.52406	0.170736
Blood urea nitrogen	25.32614	278.125	-0.25941	0.248441
Hematocrit	37.38885	35.51481	0.085771	0.266198
hemoglobin	12.26037	4.466132	0.066073	-0.57179
Lymphocytes	18.14695	118.7615	-0.15113	-0.24534
Mean corpuscular volume	91.13186	47.43263	0.024414	0.130497
Reticulocyte distribution width	14.43185	8.617955	-0.21524	0.225659
Mean heart rate	79.12643	216.1273	-0.11063	0.214461
Minimum ED systolic blood pressure	127.5979	544.2491	-0.02563	-0.34601
Supplemental oxygen	0.200931	0.160557	-0.50145	0.259794
Outpatient cardiovascular medications	0.632983	0.232315	0.76146	-0.22383
ED diagnosis of acute cerebrovascular disease	0.015183	0.014953	-0.12417	0.174096
ED diagnosis of syncope	0.024622	0.024016	-0.15888	-0.06314
intercept				-2.43121

© 2024 Haimovich AD et al. *JAMA Network Open.*

eTable 4: Missing GEST variables across the study cohort.

Variable	Number missing	Percent missing
Age	0	0%
History of secondary malignancy	0	0%
History of lung cancer	0	0%
History of delirium, dementia, or other cognitive disorders	0	0%
History of pancreatic cancer	0	0%
Number of admissions in last year	0	0%
Blood urea nitrogen	8773	10.7%
Hematocrit	8809	10.7%
hemoglobin	8814	10.7%
Lymphocytes	10322	12.5%
Mean corpuscular volume	8828	10.7%
Reticulocyte distribution width	8848	10.7%
Mean heart rate	451	0.5%
Minimum ED systolic blood pressure	476	0.6%
Supplemental oxygen	404	0.5%
Outpatient cardiovascular medications	82371	100%
ED diagnosis of acute cerebrovascular disease	0	0%
ED diagnosis of syncope	0	0%

© 2024 Haimovich AD et al. *JAMA Network Open.*

eTable 5: GEST AUROCs for all encounters vs first index encounter per patient during study period.

	All	Index Encounter
	0.79 (0.79-0.79)	0.79 (0.78-0.79)
**Race**		
BL	0.82 (0.81-0.83)	0.82 (0.79-0.84)
Asian	0.81 (0.79-0.83)	0.80 (0.76-0.83)
White	0.78 (0.78-0.79)	0.78 (0.77-0.78)
**Ethnicity**		
Hispanic/Latino	0.78 (0.75-0.80)	0.78 (0.74-0.82)
Non-Hispanic/Latino	0.79 (0.79-0.80)	0.79 (0.78-0.79)
**Sex**		
Female	0.80 (0.79-0.81)	0.80 (0.79-0.81)
Male	0.78 (0.77-0.79)	0.78 (0.77-0.79)
**Age**		
60s	0.81 (0.80-0.82)	0.80 (0.78-0.81)
70s	0.80 (0.79-0.80)	0.78 (0.77-0.79)
80s	0.74 (0.73-0.75)	0.72 (0.71-0.74)
90+	0.68 (0.67-0.70)	0.68 (0.66-0.70)

© 2024 Haimovich AD et al. *JAMA Network Open.*

eTable 6: Population demographics for model update procedures.

		Model update cohort (2017-2018)	Validation cohort (2019-2021)
n		34085	48286
Age, mean (SD)		77.0 (8.5)	76.7 (8.4)
Sex, n (%)	F	18833 (55.3)	25914 (53.7)
Ethnicity, n (%)	Not Hispanic or Latino	31170 (91.4)	43779 (90.7)
	Unknown	669 (2.0)	1204 (2.5)
	Hispanic or Latino	2246 (6.6)	3303 (6.8)
Race, n (%)	AI/AN	72 (0.2)	108 (0.2)
	Asian	1427 (4.2)	2090 (4.3)
	Black	6544 (19.2)	9831 (20.4)
	Native Hawaiian or other Pacific Islander	20 (0.1)	47 (0.1)
	Other	1300 (3.8)	2038 (4.2)
	Unknown	542 (1.6)	974 (2.0)
	White	24180 (70.9)	33198 (68.8)
ED disposition, n (%)	Admitted	18838 (55.3)	27094 (56.1)
	Home	11198 (32.9)	14971 (31.0)
	Observation then Admitted	1020 (3.0)	1629 (3.4)
	Observation then Home	3029 (8.9)	4592 (9.5)
Six month mortality, n (%)		4510 (13.2)	6878 (14.2)
Serious illnesses, n (%)		18464 (54.2)	25513 (52.8)
	Dementia, n (%)	1528 (4.5)	2207 (4.6)
	Renal failure, n (%)	931 (2.7)	1238 (2.6)
	Diabetes, n (%)	2481 (7.3)	3537 (7.3)

© 2024 Haimovich AD et al. *JAMA Network Open.*

Neurodegenerative disease, n (%)	17 (0.0)	36 (0.1)
Lung disease, n (%)	4229 (12.4)	6122 (12.7)
Heart disease, n (%)	5120 (15.0)	6640 (13.8)
Cancer, n (%)	1807 (5.3)	2597 (5.4)
Hip fracture, n (%)	219 (0.6)	293 (0.6)
Liver disease, n (%)	863 (2.5)	1188 (2.5)
Stroke/TIA*, n (%)	665 (2.0)	780 (1.6)
>= 80 yrs, n (%)	12539 (36.8)	16807 (34.8)

© 2024 Haimovich AD et al. *JAMA Network Open.*

eTable 7: Comparison between original logistic regression coefficients and updated coefficient model.

Outpatient cardiovascular medications were unavailable for this cohort and so were not included in the updated model.

	Original GEST model	Updated model
Age	0.508937	0.433034
History of secondary malignancy	0.2183	0.293718
History of lung cancer	0.138943	0.068623
History of delirium, dementia, or other cognitive disorders	0.142679	0.106543
History of pancreatic cancer	0.119607	0.025528
Number of admissions in last year	0.170736	0.03723
Blood urea nitrogen	0.248441	0.167535
Hematocrit	0.266198	-0.1799
hemoglobin	-0.57179	-0.00212
Lymphocytes	-0.24534	-0.29903
Mean corpuscular volume	0.130497	0.205825
Reticulocyte distribution width	0.225659	0.397137
Mean heart rate	0.214461	0.178287
Minimum ED systolic blood pressure	-0.34601	-0.23091
Supplemental oxygen	0.259794	0.176933
Outpatient cardiovascular medications	-0.22383	*
ED diagnosis of acute cerebrovascular disease	0.174096	0.118422
ED diagnosis of syncope	-0.06314	-0.07448
intercept	-2.43121	-2.31832

© 2024 Haimovich AD et al. *JAMA Network Open.*

eTable 8: Test characteristics of GEST as compared to serious illness diagnoses for only the first encounter per patient in the study period.

Percent of patients indicates the percentage of all encounters meeting the criteria. 95% confidence intervals are shown in parenthesis.

	Percent of patients	Sensitivity	Specificity	PPV	NPV	LR+	LR-
SI	39.30%	61.8% (60.4% - 63.2%)	63.7% (63.2% - 64.2%)	18.7 (18.0 - 19.0)	92.5 (92.0 - 93.0)	1.7 (1.6 - 1.8)	0.6 (0.57 - 0.63)
GEST >5%	46.60%	83.6% (82.5% - 84.6%)	58.4% (57.9% - 58.9%)	21.4 (21.0 - 22.0)	96.3 (96.0 - 97.0)	2.0 (1.9 - 2.2)	0.28 (0.26 - 0.3)
GEST >10%	26.00%	62.9% (61.5% - 64.3%)	79.0% (78.6% - 79.4%)	28.8 (28.0 - 30.0)	94.0 (94.0 - 94.0)	3.0 (2.8 - 3.2)	0.47 (0.44 - 0.5)
GEST >20%	11.50%	36.8% (35.4% - 38.2%)	91.9% (91.6% - 92.1%)	38.0 (37.0 - 39.0)	91.5 (91.0 - 92.0)	4.5 (4.2 - 4.8)	0.69 (0.65 - 0.73)
GEST >30%	6.10%	22.5% (21.3% - 23.6%)	96.1% (95.9% - 96.3%)	43.7 (42.0 - 46.0)	90.2 (90.0 - 90.0)	5.7 (5.3 - 6.2)	0.81 (0.74 - 0.87)

© 2024 Haimovich AD et al. *JAMA Network Open.*

Supplementary References

1. Janssen KJM, Vergouwe Y, Kalkman CJ, Grobbee DE, Moons KGM. A simple method to adjust clinical prediction models to local circumstances. *Can J Anaesth*. 2009;56(3):194-201.

2. Haimovich AD, Xu W, Wei A, Schonberg MA, Hwang U, Taylor RA. Automatable end-of-life screening for older adults in the emergency department using electronic health records. *J Am Geriatr Soc*. Published online February 6, 2023. doi:10.1111/jgs.18262

3. Walling AM, Sudore RL, Bell D, et al. Population-Based Pragmatic Trial of Advance Care Planning in Primary Care in the University of California Health System. *J Palliat Med*. 2019;22(S1):72-81.

4. Kelley AS, Ferreira KB, Bollens-Lund E, Mather H, Hanson LC, Ritchie CS. Identifying Older Adults With Serious Illness: Transitioning From ICD-9 to ICD-10. *J Pain Symptom Manage*. 2019;57(6):1137-1142.

5. Curtis JR, Lee RY, Brumback LC, et al. Intervention to Promote Communication About Goals of Care for Hospitalized Patients With Serious Illness: A Randomized Clinical Trial. *JAMA*. 2023;329(23):2028-2037.

© 2024 Haimovich AD et al. *JAMA Network Open.*
