# Supplementary material for: Geriatric End-of-Life Screening Tool Prediction of 6-Month Mortality in Older Patients
Source: JAMA Netw Open. 2024 May 31;7(5):e2414213. doi: 10.1001/jamanetworkopen.2024.14213 (PMC11143461; doi:10.1001/jamanetworkopen.2024.14213)
Supplement: Supplement 3. — Data Sharing Statement [file jamanetwopen-e2414213-s003.pdf]

# Data Sharing Statement

Haimovich. Geriatric End-of-Life Screening Tool Prediction of 6-Month Mortality in Older Patients. *JAMA Netw Open*. Published May 31, 2024.
doi:10.1001/jamanetworkopen.2024.14213

**Data**
**Data available:** No
